# Delphi consensus finding on paediatric-adult transition: results from the epilepsy transition working group of the italian league against epilepsy (LICE)

**DOI:** 10.1007/s10072-025-08166-y

**Published:** 2025-06-04

**Authors:** F. Darra, P. Biermann Klaus, D. Audenino, F. Bisulli, A. Cossu, M. Elia, A. La Neve, M. Mancardi, C. Pastori, J. Proietti, F. Ragona, R. Rizzi, A. Rosati, V. Sciruicchio, N. Specchio, C. Stipa, C. Zanus, F. Villani

**Affiliations:** 1 Unit of Child Neuropsichiatry, Azienda Ospedaliera Universitaria Integrata of Verona, Italy (Full Member of European Reference Network EpiCARE), Verona, Italy; 2 Department of Innovation Medicine and Engineering, University of Verona, Verona, Italy; 3 Department of Health Professions, Meyer Children’S University Hospital, Florence, Italy; 4 Department of Geriatric, Neurology Unit, Neurological and Rehabilitation Sciences, E.O OspedaliGalliera, Genova, Italy; 5 Department of Biomedical and Neuromotor Sciences, University of Bologna, Bologna, Italy; 6 Unit of Neurology and Clinical Neurophysiopathology, Oasi Research Institute-IRCCS, Troina, Italy; 7 DiBraiN Department, University of Bari Aldo Moro, Bari, Italy; 8 Department of Neuroscience, Rehabilitation, Ophthalmology, Genetics, Maternal and Child Health (DINOGMI), University of Genoa, Genoa, Italy; 9 Epilepsy Unit, Fondazione IRCCS Istituto Neurologico Carlo Besta, Milan, Italy; 10 Department of Paediatric Neuroscience, European Reference Network EpiCARE, Fondazione IRCCS Istituto Neurologico Carlo Besta, Neurology Unit, Milan, Italy; 11 Department of Neuro-Motor Diseases, Azienda Unità Sanitaria Locale - IRCCS of Reggio , Emilia, Italy; 12 Child Neuropsychiatry Unit, Pediatric Department, Santa Chiara Hospital, Azienda Provinciale Per I Servizi Sanitari (APSS), Trento, Italy; 13 Children Epilepsy and EEG Center, PO San Paolo ASL, Bari, Italy; 14 Neurology Epilepsy and Movement Disorders Unit, Full Member of European Reference Network on Rare and Complex Epilepsies, Bambino Gesu’ Children’s Hospital, IRCCS, EpicARE, Rome, Italy; 15 IRCCS Istituto Delle Scienze Neurologiche Di Bologna (Reference Center for Rare and Complex Epilepsies - EpiCARE), Bologna, Italy; 16 Institute for Maternal and Child Health IRCCS Burlo Garofolo, Trieste, Italy; 17 Division of Clinical Neurophysiology and Epilepsy Centre, IRCCS Ospedale Policlinico San Martino, Genoa, Italy

**Keywords:** Epilepsy, Transition, Multidisciplinary care, Delphi consensus, Developmental disabilities, Tailored care

## Abstract

**Purpose and rationale:**

The care for paediatric and transitional-age epilepsy patients has expanded significantly, addressing the diverse needs of patients with self-limiting to lifelong conditions. Approximately one-third of patients with childhood-onset epilepsy remain dependent on parental care, with transition influenced by factors such as seizure frequency, drug resistance, comorbidities, and developmental disabilities. The Italian League Against Epilepsy (LICE) initiated a Delphi consensus to establish common guidelines for effective transition practices.

**Methods:**

The consensus process included a literature review, thematic analysis, and iterative surveys using the Delphi Technique. The surveys involved 15 clinicians from LICE centres, forming the Epilepsy Transition Working Group (ETWG), and external experts. The surveys gathered expert opinions, with questions designed from evidence-based thematic areas.

**Results:**

The Delphi rounds revealed several findings. Effective pediatric-to-adult transition in epilepsy requires a multidisciplinary approach, including child and adolescent neuropsychiatrists, continuous training for healthcare providers, and active involvement of caregivers. The transition process should start at variable ages depending on the type of epilepsy and associated comorbidities, and it should mitigate risk factors and address psychological stress for patients and caregivers. Items related to transition tools did not reach consensus, highlighting the need for standardized screening questionnaires and measurable outcomes.

**Conclusions:**

This study emphasizes the necessity of an organized transition model involving various specialists and a tailored timeline. The consensus underscores the importance of caregiver involvement and unified educational curricula for all epileptologists to ensure effective care transition. The ETWG is building and improving a network of epilepsy centres to implement the organizational model derived from this study.

## Introduction

Over the first ten years of this century, care for patients with epilepsy of paediatric and transitional age has expanded [1]. While some paediatric patients present with self-limiting and age-related conditions, other patients will continue to need periodic neurological evaluations and social support throughout their adult lives, but around one-third of patients with childhood-onset epilepsy fail to transition to independence from parental care [2, 3].

The transition process is influenced by different factors, among which are seizure frequency, specific epileptic syndromes, possible drug resistance, the need for non-pharmacological treatments, and the presence of multimorbidities [4]. Young People with Epilepsy (YPWE) with moderate to severe developmental disability report lower overall education, which can hinder their transition to adulthood [5]. In this landscape few examples of a practical and functional transition process exist [6].

Currently, in Italy, different organizational models for the transition have been proposed, among them regional Diagnostic and Therapeutic Care Pathways (Pecorsi Diagnostici Terapeutici Assistenziali, PDTA) have been developed. However, with few isolated exceptions, none have been adopted successfully and shared nationwide. The main difficulty is related to the fact that the Healthcare System (HS) is divided into twenty different, independent regional systems.

Furthermore, other peculiar aspects of the Italian HS make implementing recommendations from international literature difficult. For example, the peculiar organization of children’s epileptology services, making use of both Paediatricians and Neuropsychiatrists, a figure almost exclusive to the Italian landscape.

The Italian League Against Epilepsy (LICE), to define common guidelines for the best transition methods, has initiated a Delphi consensus to identify the factors that may influence the success or failure of the transition process involving all LICE centres across the country.

## Methods

**Fig. 1:**
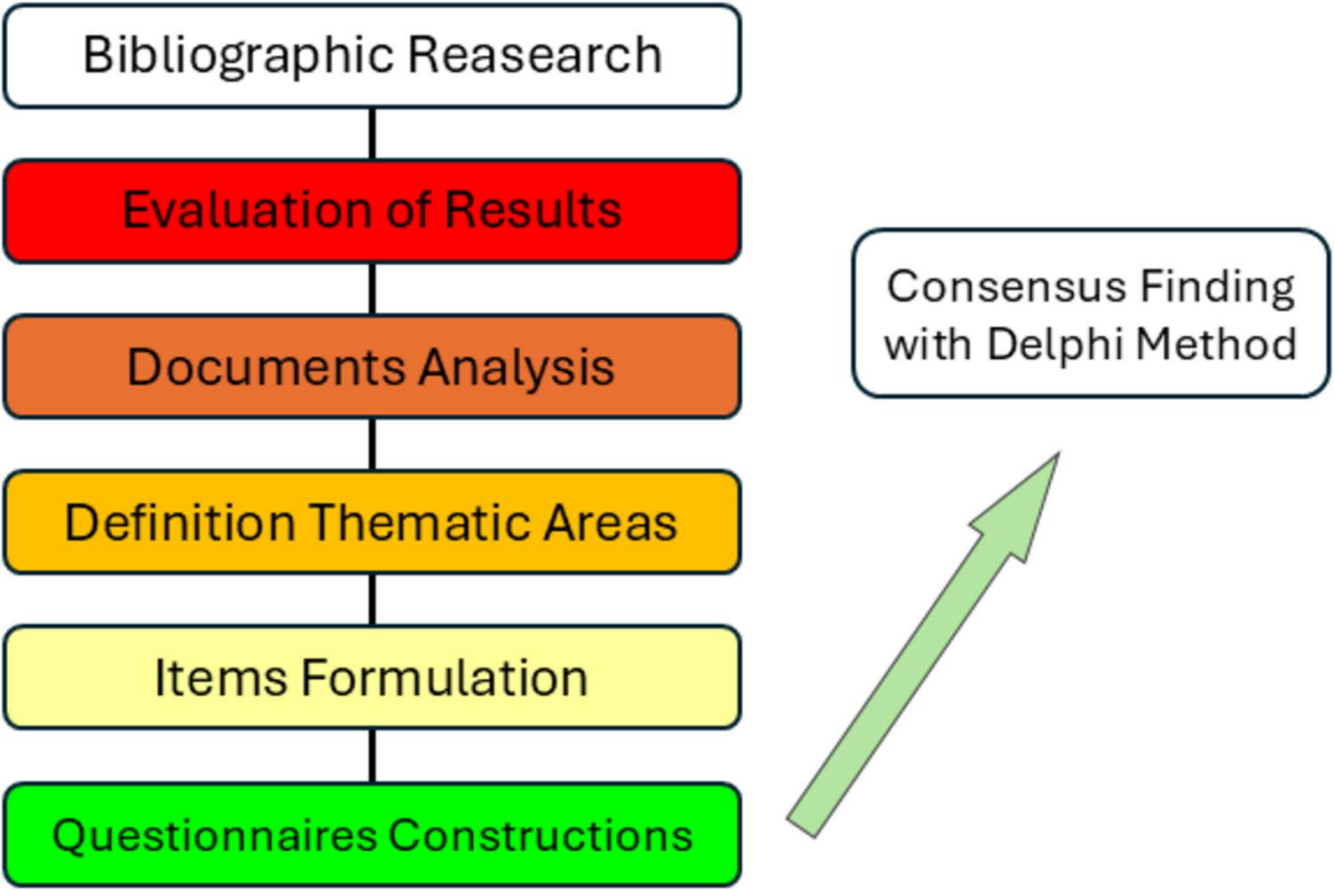
Flowchart graph depicts the steps undertaken by the Working Group to extract the relevant concepts from literature and translate them into items grouped in thematic areas, which are then used in the Delphi Consensus Method

The methodological approach of our consensus-finding process (see Fig. 1) consisted of conducting a literature review and subsequent evaluation of the results by the Epilepsy Transition Working Group (ETWG), including the analysis of the selected studies, defining thematic areas, formulating questions, and conducting an iterative survey using the Delphi Technique.

The literature search was conducted on the PubMed, Embase, Cochrane, and Scopus search engines (last consultation date 03/08/2021) using the following search strings:

- ((transfer OR transition) AND (service OR care OR clinic OR issue OR model OR program)) OR"Transitional Care"[Mesh] OR"Transition to Adult Care"[Mesh]
- (epilepsy OR epileptic)
- (adolescence OR adolescent OR teen OR teenager OR young adult)
- #1 AND #2 AND #3
- #1 AND #2 Filter: Adolescent, Young Adult
- #4 OR #5 Filter: English, Date of publication: 2001–2020

**Fig. 2:**
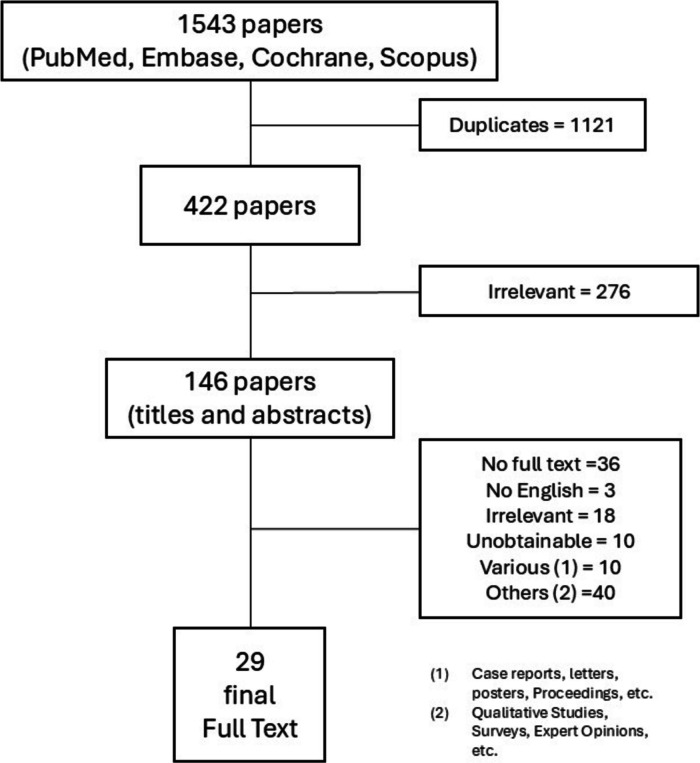
Flowchart depicting the exclusion and inclusion steps of the papers analysed in the literature review

The evaluation of the search results returned 1543 articles. After eliminating duplicates, irrelevant works results without full text, those in languages other than English, unobtainable papers, and excluding case reports, letters, congress posters and proceedings, survey-based studies, expert opinions, and qualitative studies,, 29 articles remained for examination (Fig. 2).

The analysis of the included works, which encompassed publications of observational studies and some literature reviews, was conducted using the Evidence Tables provided in Appendix H of the NICE Manual for Guideline Development [7].

The study of international scientific literature that formed the basis of the consensus-finding process identified the 13 domains or thematic areas listed in Table 1. The articles were assigned to each thematic area based on the Evidence Tables, providing a concise analytical description and the treated topic. This step generated 28 items between questions and statements to be submitted to the Expert Panel (EP) for evaluation, listed in Table 2.

**Table 1:** Reporting all 13 thematic areas emerged from literature review

Thematic areas emerged from literature review
1. Conceptual Model (e.g., defining the goals of transition sufficiently well-defined in the literature)
2. Organizational Model (e.g., understanding the tools available for better organization, e.g., integrated multidisciplinary clinics, resources to be utilized, etc.)
3. Training (e.g., training operators with standard courses for child neuropsychiatric and adult neurologists but also general practitioners, social workers, nurses, etc.)
4. Tools (e.g., telemedicine, questionnaires, spaces, apps, etc.)
5. Performance (e.g., defining indicators for outcome evaluation)
6. Risk Factors (e.g., case complexity for intellectual disabilities, social distress, geographic difficulties, fear of abandonment, etc.)
7. Barriers (e.g., inadequate spaces in waiting rooms, clinics, the possibility of regular hospitalization in a dedicated room with the caregiver allowed to stay, comorbidities, intellectual disabilities, emotional barriers, etc.)
8. Failure (e.g., identifying reasons for failure for a correct transition
9. Improvement (e.g., the possibility of introducing corrections after an audit of the process and involving the territorial management of the patient (General Practitioner))
10. Impact (e.g., positive impacts on the patient and caregiver, as well as on the optimization of resources)
11. Development (e.g., integration into the chronic care pathway and the implementation of specialized networks not connected)
12. Caregiver (e.g., caregivers play essential roles in the transition process both for the effectiveness of therapeutic interventions and for the quality of life of the patient)
13. Gender (e.g., considering gender differences in transition planning)

**Table 2:** Listing items submitted to the Expert Panel for consensus in all Core Criteria Set step. Reporting 28 items in CCS-1, 19 items in CCS-2, and 11 in CCS-3

CCS- 1 items
1. Transition is a process planned and prepared over time by the child neuropsychiatrist alone
2. Transition is a handover from the child neuropsychiatrist to the adult neurologist
3. The approach of the adult neurologist is less holistic than that of the child neuropsychiatrist
4. Multidisciplinary management is the foundation of an effective transition process
5. How do you assess the need for a conceptual transition model for managing epilepsy?
6. Should an organizational transition model be the same for different forms of epilepsy?
7. Should an organizational transition model be diversified for various forms of epilepsy?
8. When implementing an efficient transition model, how do you consider primary training events and ongoing update for operators?
9. What assessment tools does the operator use on the person with epilepsy during the transition?
10. Are there self-assessment tools for the person with epilepsy or caregiver during the transition?
11. Should self-assessment tools be diversified by age group if applicable?
12. Should self-assessment tools be diversified by type of epilepsy (relative to intellectual disability)?
13. How do you evaluate the use of the following questionnaires to verify the effectiveness of the transition process?
14. Satisfaction questionnaires?
15. Questionnaires on the quality of life of the adult patient?
16. Interventions established during the transition can influence the outcome of individuals with epilepsy
17. Good effectiveness during the transition process is closely related to the presence of a multidisciplinary team in the adult epilepsy center, which includes Child and Adolescent Neuropsychiatrists
18. Interventions established during the transition can reduce the risk factors for individuals with epilepsy
19. The simultaneous presence of physical or intellectual disabilities constitutes a barrier to the transition of individuals with epilepsy
20. The simultaneous presence of socioeconomic barriers can significantly influence the transition
21. Failure in epilepsy transition can worsen the prognosis in terms of social consequences (e.g., quality of life, employment inclusion, independence)
22. The role of the caregiver in the transition process is essential for managing epilepsy in terms of favorable outcomes
23. The transition process is a psychological stress factor for the patient and the caregiver
24. Non-compliance or resistance of parents/caregivers to the transition process can be a source of failure in the transition, especially for patients with disabilities
25. Gender conditions the effectiveness of the epilepsy transition process
26. The possibility of a sexual/reproductive life may influence the age at which to initiate the transition process
27. Is there a defined age for transition?
28. Should the age for transition consider the type of epilepsy or different comorbidities?
CCS- 2 items
1. The transition is generally initiated by the child neuropsychiatrist who follows the patient with epilepsy
2. The transition is not merely a handover from the child neuropsychiatrist to the adult neurologist
3. The organizational model for transition should have some differences based on the various forms of epilepsy
4. The simultaneous presence of physical or intellectual disabilities could act as a barrier to the effective transition of people with epilepsy
5. Gender should not affect the effectiveness of the transition
6. The age at which the transition process is initiated could be influenced by sexual/reproductive life
7. The age at which the transition process begins may vary based on different factors
8. In most cases, the approach of the adult neurologist is less holistic than that of the child neuropsychiatrist
9. A conceptual model of transition is an important starting point for sharing care pathways for patients with epilepsy
10. It is preferable that the organizational model for transition be differentiated for the various forms of epilepsy
11. Would you consider it useful to use evaluation tools by the operator on the person with epilepsy during the transition to verify its success?
12. Would you consider it useful to use self-assessment tools for the person with epilepsy and the caregiver to verify the success of the transition?
13. If self-assessment tools are used, would you find it useful to ensure they are differentiated by age group to verify the success of the transition?
14. If self-assessment tools are used, would you find it useful to ensure they are differentiated by the type of epilepsy?
15. Do you consider it useful to use questionnaires to verify the effectiveness of the transition process?
16. Do you consider it useful to use questionnaires to verify satisfaction with the transition process?
17. Do you consider it useful to use questionnaires to verify the quality of life of the patient and the caregiver, both in childhood and adulthood?
18. Interventions implemented during the transition could potentially reduce risk factors for people with epilepsy
19. Could the presence of socio-economic issues affect the transition process?
CCS- 3 items
1. The simultaneous presence of physical or intellectual disabilities could act as a barrier to the effective transition of people with epilepsy
2. The age at which the transition process is initiated could be influenced by sexual/reproductive life
3. In most cases, the approach of the adult neurologist is less holistic than that of the child neuropsychiatrist
4. Would you consider it useful to use evaluation tools by the operator on the person with epilepsy during the transition to verify its success?
5. Would you consider it useful to use self-assessment tools for the person with epilepsy and the caregiver to verify the success of the transition?
6. If self-assessment tools are used, would you find it useful to ensure they are differentiated by age group to verify the success of the transition?
7. If self-assessment tools are used, would you find it useful to ensure they are differentiated by the type of epilepsy?
8. Do you consider it useful to use questionnaires to verify the effectiveness of the transition process?
9. Do you consider it useful to use questionnaires to verify satisfaction with the transition process?
10. Do you consider it useful to use questionnaires to verify the quality of life of the patient and the caregiver, both in childhood and adulthood?
11. Could the presence of socio-economic issues affect the transition process?

**Fig. 3:**
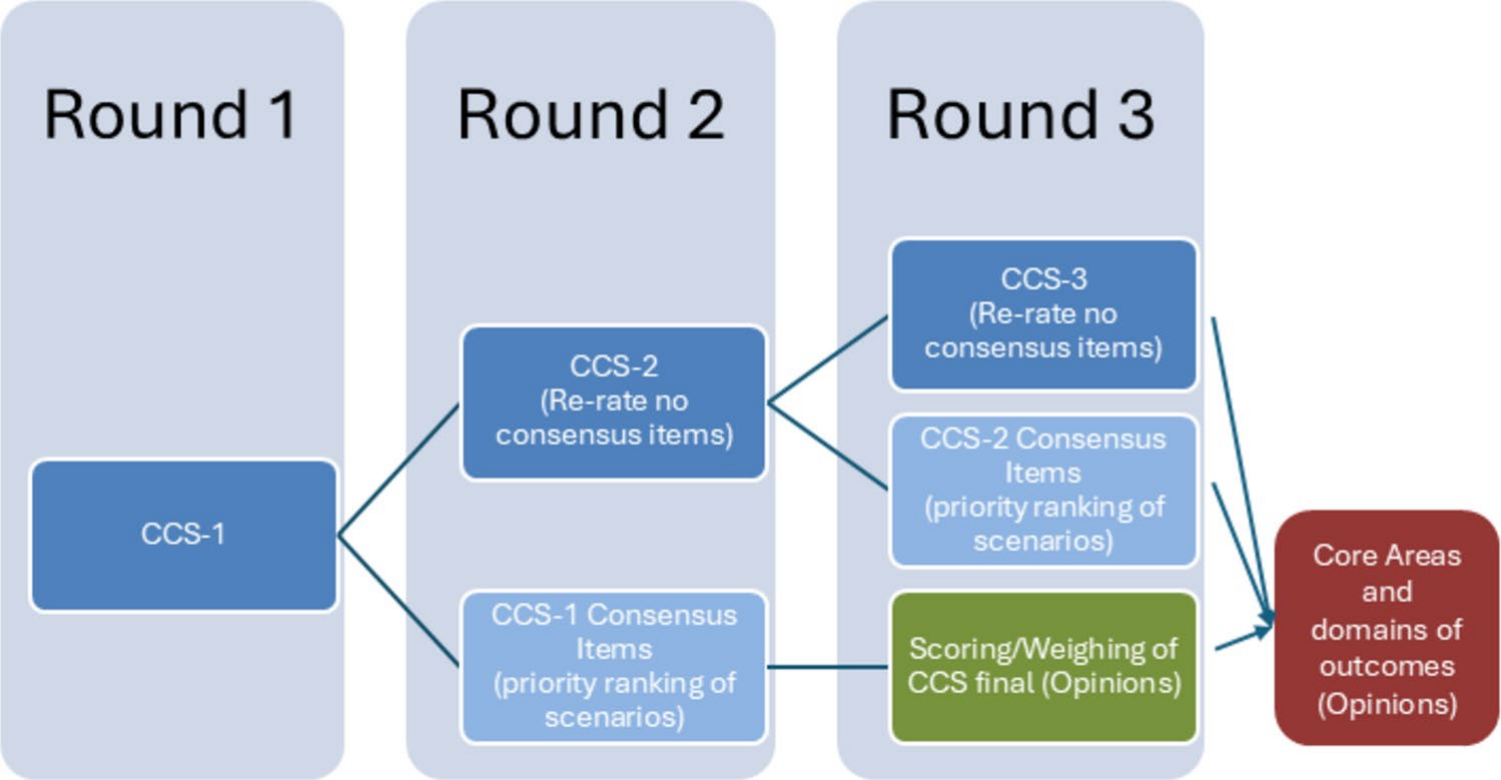
Flowchart depicting the Delphi Methods steps

The rules for managing the COVID- 19 pandemic period, the distribution of LICE Centres throughout the national territory, and the high number of experts involved in the consensus finding process (representatives from the 50 LICE Centres and external experts) indicated the use of the Delphi Method, summarized in Santaguida’s work[8], schematically represented in Fig. 3.

The Delphi Technique iterative survey participants were 15 clinicians from various LICE Centres nationwide, forming the ETWG and acting as the Steering Committee (SC), comprising 7 paediatric epileptologists and 8 adult epileptologists. The Facilitator was a neutral individual with expertise in research methodology and data collection. The EP consisted of clinicians affiliated with LICE Centres and non-affiliated clinicians. In the final phase of Delphi, 13 Stakeholders joined, including eight clinicians from other relevant specialties and five members of Patients Advocacy Groups.

The EP received a questionnaire containing all the questions/items (Core Criteria Set 1, CCS- 1) formulated by the ETWG in the first Delphi Round. The experts were asked to provide their assessments to obtain a broad view of the experts’ opinions, aiming to frame the topic and draw an overall picture of the investigated problem. They were also encouraged to add the rationale for their responses and any observations related to the items.

The 2nd Delphi Round (CCS- 2) questionnaire reformulated all items near and far from consensus. In the 3rd Delphi Round (CCS- 3) questionnaire, the remaining items near and far from consensus were revisited after extensive discussion during an in-person and online meeting on April 19 th, 2023.

The questionnaires were developed on Google Workspace using the Forms version and were accessible only to the Panel members. Responses to the items/questions were mandatory on an odd-numbered Likert scale with assigned ratings, and optional free-text observations.

The ETWG defined the cut-off for consensus at 75% of responses and items when either included or excluded from the final Core Criteria Set (CCS). Data processing considered the sum of Likert values 1 + 2, 4 + 5, and the neutral value of 3 assigned to each item. Consensus for exclusion was considered for an item when the sum of Likert values 1 + 2 was ≥ 75%, while consensus for inclusion was considered when the sum of Likert values 4 + 5 was ≥ 75%. Items formed the basis for subsequent Delphi Rounds when they had values of 3 ≥ 75% or the sum of Likert values 4 + 5 < 75%. The responses to the questionnaires from the three Delphi Rounds were processed as aggregated data and as stratified data for adult neurologists and child neuropsychiatrists.

Additionally, the rationale for the response on the Likert scale and free-text observations were analysed when available (742 for CCS- 1, 453 for CCS- 2).

At the end of the third Delphi Round, a final questionnaire was administered to the EP and Stakeholders. The provided responses were aimed at:

- Defining the main areas for immediate implementation and their related outcome indicators
- Establishing the period for the initial application
- Scheduling the time for evaluating the outcomes of the initial application and revising the document.

The EP was requested to assign a Value to each thematic area on a scale from 0 (not important) to 10 (very important) and a Weight (i.e., if its contextualization has priority or not) on a scale from 1 (not a priority) to 5 (a priority). For the analysis of the assigned Value, values from 0 to 3 have been grouped into the category ‘less important’, values from 4 to 6 in the category ‘important’, and values from 7 to 10 in the category ‘very important’. The assignment of Weight expresses a judgment on the priority of application of the respective domain. Data related to weight have been grouped into three categories for easier reading: less prioritized (1–2), moderately prioritized (3), and prioritized (4–5).

A similar approach was used for each item resulting from the Delphi questionnaires, to which the EP assigned a Value, a Weight, and a Timing of implantation on a scale from 1 (not urgent) to 5 (very urgent). Additionally, for each item, they were required to provide an outcome indicator and were allowed to add comments.

Required tasks included defining ‘Transition’ as the American Academy of Pediatrics (AAP) [3] provided, identifying outcome indicators for organizational model items, and offering feedback on local contextualization and timelines on the benchmarking and the final document’s revision.

## Results

### Results from CCS- 1

The CCS- 1 questionnaire was sent to 70 experts comprising the panel, 50 LICE Centers, and 20 independent experts not associated with LICE. Sixty-one responses were received, showing excellent responsiveness at 87.2% (44/50 LICE Centers I-III level, 88%, and 17/20 Others 85%).

Experts strongly agreed on 32.1% of the proposed items.

At the end of this first step, the following questions can be identified as common viewpoints:

- An effective paediatric-to-adult transition process in epilepsy care is based on the multidisciplinary management of the patient [92%].
- Good effectiveness during the paediatric-to-adult transition process in epilepsy care is closely related to the presence of a multidisciplinary team in the adult epilepsy center, including Child and Adolescent Neuropsychiatrists [84%].
- Basic training and continuous updates for operators are essential to implement an efficient paediatric-to-adult transition model in epilepsy care [77%].
- Interventions established during the paediatric-to-adult transition process influence the outcomes of individuals with epilepsy [87%].
- Failure in the paediatric-to-adult transition process in epilepsy care can worsen the prognosis regarding social consequences (e.g., quality of life, employment inclusion, independence) [93%].
- The role of the caregiver in the paediatric-to-adult transition process is essential for managing epilepsy in terms of favourable outcomes [95%]. Non-compliance or resistance of parents/caregivers to the paediatric-to-adult transition process in epilepsy care can be a source of failure in the transition (especially for patients with disabilities) [93%].
- The paediatric-to-adult transition path in epilepsy care should be considered a factor of psychological stress for the patient and the caregiver [82%].
- The age at which to activate the paediatric-to-adult transition process should consider the type of epilepsy and different comorbidities [75%].

### Results from CCS- 2

The CCS- 2 questionnaire was sent to 84 experts, 50 LICE Centers at Levels I to III, and 35 other independent experts not associated with LICE. Fifty-eight responses were received, with a response rate of 69% (37/50 LICE Centers I-III level, 74%, and 23/34 Others 68%).

Consensus of the Panel was obtained for 42% of the 19 re-proposed items, for 60.7% consensus on 28 items from the starting point (CCS- 1).

At the end of this second step, the following common viewpoints can be identified in addition to those already reported for CCS- 1:

- A conceptual transition model is an important starting point for sharing care paths for patients with epilepsy [89.7%].
- The transition is initiated by the child neuropsychiatrist who follows the patient with epilepsy [93%].
- Considering that the transition is not a simple handover from the child neuropsychiatrist to the adult neurologist, it is necessary to develop and share a specific procedure at the highest possible level [89.7%].
- The organizational transition model must consider differences related to different forms of epilepsy and, consequently, must be diversified for various forms of epilepsy [96.6% and 79.3%; 2 items merged into a single question].
- The effectiveness of the transition should not be conditioned by the gender of the patient [93.1%].
- The age at which the transition begins varies based on different factors [89.7%].
- Interventions established during the transition are also aimed at reducing risk factors for individuals with epilepsy [81%].

### Results from CCS- 3

The CCS- 3 questionnaire was administered in real-time during a blended meeting on April 19 th, 2023. Within a week, 48 experts from the Panel responded. A final consensus of the Panel was obtained at 68% on 28 items.

At the end of this third step, the following common viewpoints can be identified in addition to those already reported for CCS- 1 and CCS- 2:

- In most cases, the approach of adult care services is less holistic than that of the paediatric age, and there is no multidisciplinary structured setting like that of the child neuropsychiatry [79%].
- The use of patient and caregiver quality-of-life questionnaires is helpful for a longitudinal assessment of patients before and after the transition process [75%].

After three rounds, the following items did not find consensus:

- The simultaneous presence of physical or intellectual disabilities might constitute a barrier to the effective transition of individuals with epilepsy [70.8%] – Thematic area “Barriers”.
- The age at which to decide to initiate the transition process might be conditioned by sexual/reproductive life [62.5%] – Thematic area “Gender”.
- Would you find the use of assessment tools on the person with epilepsy helpful in assessing the success of the transition process? [52.1%] – Thematic area “Tools”.
- Would you find the use of self-assessment tools helpful for assessing the success of the process? [64.6%] – Thematic area “Tools”.
- In the case of using self-assessment tools, would you find it helpful if they were diversified by age group? [50%] – Thematic area “Tools”.
- In the case of using self-assessment tools, would you find it helpful if they were diversified by type of epilepsy? [43.75%] – Thematic area “Tools”.
- Do you consider the use of questionnaires useful to verify the effectiveness of the transition process? [70.8%] – Thematic area “Tools”.
- Do you consider questionnaires useful to assess satisfaction with the transition process? [70.8%] – Thematic area “Tools”.
- Could the presence of socio-economic issues condition the transition process? [64.6%] – Thematic area “Barriers”

**Fig. 4:**
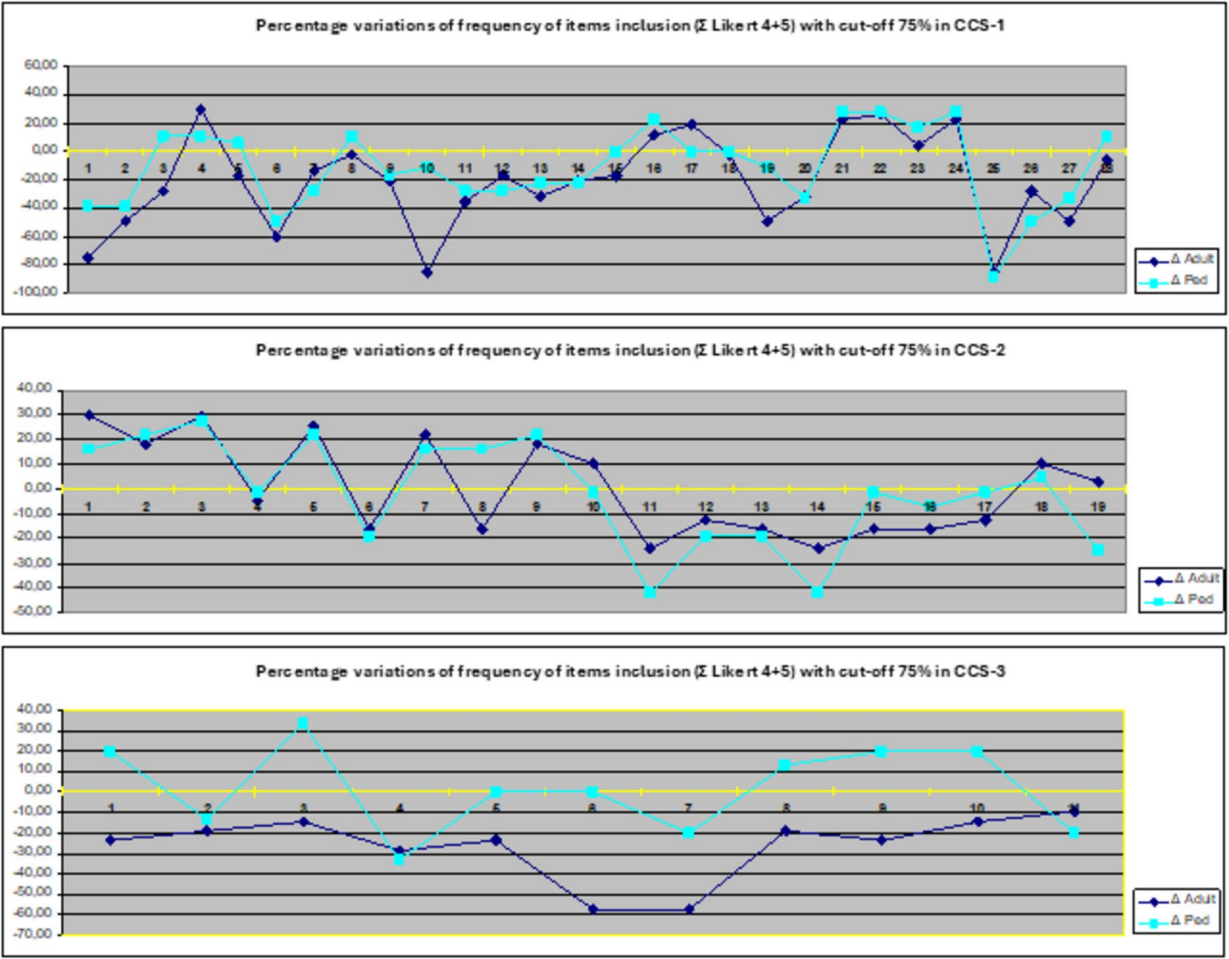
Graphs depicting the percentage variation (Δ) in the frequency of items inclusion (Σ Likert 4–5) of CCS questionnaire items, when the percentage was divided in Adult epileptologists (Adult) and Paediatric epileptologists (Ped). The items in the three graphs are numbered as reported in Table 2

In reaching the consensus emerged a significant difference between the figures involved with paediatric and adult patients. This difference was greatly evidenced by the percentage variation (Δ%) in the frequency of inclusion (∑ Likert 4–5) of CSS questionnaire items, when the percentage was divided in adult epileptologists (Adult) and Paediatric epileptologists (Ped) (Figure 4).

### Result of the final Consensus Finding Questionnaire through the Delphi Method.

Eighty-five responses were received from the EP, provided by 37 paediatric clinicians, 35 adult neurologists, and 13 stakeholders (8 non-neurologist clinicians and five representatives of patient advocacy Groups).

The results from the Value assignment show the following domains as important to very important, in descending order: Organizational Model, Training, Caregiver, Development, Tools, Barriers, Risk Factors, Improvement, Outcomes, Conceptual Model, Failure, and Performance. The domain of Gender is attributed to a relatively low Value.

Similarly, the domain of Gender is assigned little Weight. At the same time, applying the Organizational Model is considered a priority, followed by Training, Caregiver, Conceptual Model, Tools, Barriers, Development, Risk Factors, Improvement, Outcomes, Performance, and Failure.

Concerning the agreement of the definition of transition, 61.2% fully agree with the definition provided by the AAP [3], 35.3% partially agree, and only 3.5% disagree. In most cases, disagreement or partial agreement refers to the wording'…that lasts a lifetime,'which is considered unacceptable.

### Organizational Model

Regarding the organizational model, the EP found broad consensus on the identification of these eight outcomes:

1. The organizational transition model should consider differences related to various forms of epilepsy and, consequently, should be diversified for different forms of epilepsy.
  ⚬ A dedicated Working Group should define the forms of epilepsy for which it is necessary to diversify the organizational model.
2. A good effectiveness during the paediatric-to-adult transition in epilepsy care is closely related to a multidisciplinary team in the adult epilepsy center, including child and adolescent neuropsychiatrists.
  ⚬ Establishment of the multidisciplinary team.
3. Basic training and continuous updating of operators are essential to implement an efficient paediatric-to-adult transition model in epilepsy care.
  ⚬ Definition of a basic training program and continuous updating.
4. The role of the caregiver in the paediatric-to-adult transition process is essential for managing epilepsy in terms of favourable outcomes.
  ⚬ Definition of the skills to be developed in caregivers.
5. The paediatric-to-adult transition path in epilepsy care should be considered a psychological stress factor for the patient and caregiver.
  ⚬ Development of a psychological stress management system (interventions, tools, etc.).
6. The age at which to activate the paediatric-to-adult transition process should consider the type of epilepsy and various comorbidities.
  ⚬ Development of general guidelines for initiating the transition process.
7. Considering that the transition is not a simple handover from the NPI to the adult neurologist, developing and sharing a specific procedure at the highest possible level is necessary.
  ⚬ Development of a procedure/a diagnostic-therapeutic-care pathway based on existing documents in some local realities.
8. The interventions set during the transition are also aimed at reducing the risk factors for people with epilepsy.
  ⚬ Analysis and classification of risk factors attributable to the transition process.

Regarding the Value and Weight of these outcomes, the creation of a multidisciplinary team was regarded as very important and an absolute priority, along with the definition of a basic training program and continuous update, the identification of the skills to be developed in the caregiver, a dedicated working group to define the forms of epilepsy for which it is necessary to diversify the organizational model, the development of a diagnostic-therapeutic-care pathway, and the development of general guidelines for initiating the transition process. Other items were found to be moderately important and a relative priority, particularly the Development of a psychological stress management system (interventions, tools, etc.) and the analysis and classification of risk factors attributable to the transition process.

Regarding the timing of implementation of these outcomes, the urgency is reported differently from the Value and Weight; in particular, developing a diagnostic-therapeutic-care pathway is regarded as urgent as creating a multidisciplinary team. Other outcomes considered as very urgent were the definition of a basic training program and continuous updating, the definition of the skills to be developed in the caregiver, the development of general guidelines for initiating the transition process, and a dedicated working group must define the forms of epilepsy for which it is necessary to diversify the organizational model. The analysis and classification of risk factors attributable to the transition process are considered non-urgent, as well as the development of a psychological stress management system.

### Contextualization, benchmarking, and document revision

The statements reported in the"Organizational Model"chapter, derived from international scientific literature, adapted to the Italian context, and that have obtained the EP’s consensus, require local contextualization. 21.2% suggest implementing contextualization in a representative number of LICE Centers, 24.7% only in LICE Centers that declare their availability, while 54.1% believe contextualization should occur in all LICE Centers.

Once contextualization has occurred, 44.7% find it useful for the Organizational Model to be applied for 18 months before initiating a national-level comparison of the outcomes observed in participating centers, 47% for 12 months, and 8.3% for six months. 28.2% believe that the final document’s review should occur within six months, 32.9% within a year, and 38.9% within 18 months after the comparison and analysis of results have occurred.

## Discussion

This study reports results from a Delphi consensus protocol involving numerous Italian Epilepsy Centers. The items subject of the Delphi consensus have been formulated based on a wide literature review and classified in 13 thematic areas and summarised in 28 statements and questions.

From the statements for which a consensus has been reached during the three stages different considerations can be made. The transition process from paediatric to adult care should be a diachronic process often requiring an extended period of time to be effective. From literature emerges the necessity for a structured and evidence-based transition to adult care, a long-lasting process that often starts at diagnosis [9]. The EP agreed that there is no fixed age for starting the process, which needs to be initiated by the paediatric epileptologist [9]. The goal of this process should be to empower YPWE to self-manage their own condition [10, 11].

A consensus was reached among experts in the “Barrier” domain, regarding specific features of the patients influencing the initiation and the successful completion of the transition process. From these statements various elements emerge as determining factors:

- The type of epilepsy and the presence of comorbidities influences the age at which the process starts,
- experts agreed that gender should not influence the completion of the process but did not reach a consensus on the necessity of modulating the age of transition based on the YPWE’s gender.

From literature, however, some intrinsic characteristics of each YPWE further influence the transition process. As a population, YPWE have a higher social stigma, preventing them from achieving their inspirational goals [12]. Among them, women are more impacted and more prone to report anxiety and mood deflection compared to men [13]. These issues in themselves harm the transition process and YPWE’s Quality of Life [14, 15]. Psychological development also impacts transition; adolescents with higher development skills and independence show higher transition readiness [16].

Constant differences were noted in responses among paediatric epileptologist and those involved in adult care, across all three consensus stages. This could be due to various factors differentiating the two specialties. Survey-based studies have found that residents in neurology have scarce education in the field of transition and are less comfortable caring for patients with disabilities compared to their peers in paediatric care [17]. Furthermore, most rare diseases with epilepsies have their onset in a very young age and specifically to the Italian landscape, adult neurology often lacks an integrated multidisciplinary setting, as agreed via the Delphi consensus, further limiting the holistic approach to care compared to paediatric epileptologists, where teams include multiple specialists dedicated to psychosocial and developmental aspects.,. This could impact the view on the transition process, as mental health issues are common in young people with epilepsy and have a negative impact on Quality of Life, independently from seizures [18].

The necessity of a multidisciplinary approach has emerged across different statements and various domains.

On the other hand, these differences highlight the necessity of an effective transition organizational model, in the adult setting in fact, patients are expected to be independent in managing the drug regimen and the relationship with their neurologist. This issue should be addressed with specific self-management education programs [11].

A further paramount factor in the success of the transition process was identified in the patients’ caregivers. In various items the consensus was reached regarding the essential role of caregivers, and the necessity to avoid resistance and seek an alliance between caregiver and healthcare provider. From the available literature in particular YPWE with an unsupportive family environment or lower IQ had a higher risk of failing the transition process [19]. The influential role of caregivers is a more profound impact in the case of patients with Developmental and Epileptic Encephalopathies considering their dependency on caregivers [20]. However, fewer transition programs are tailored to their needs [21], which results in a high percentage of patients failing to transition to an adult healthcare provider [22].

Regarding the statements for which a consensus was not reached in any of the three stages, the majority pertained the thematic area of “Tools”. The lack of consensus reflects the heterogeneity of available screening questionnaires for transition readiness, possible influencing factors leading to failure. This uncertainty in the use and application of existing tools is mirrored in the lack of questionnaires and scores to determine a single patient’s transition readiness and specific and measurable outcomes for an effective transition are lacking [23, 24].

## Conclusions

This study reports results from a Delphi consensus involving a wide number of epileptologists in the Italian community within and outside LICE, involved in paediatric and adult care other than other specialists and stakeholders.

The consensus identified as influencing factors in the transition process the involvement of different specialists and healthcare providers, a tailored timeline dictated by the type of epilepsy and existing comorbidities, taking into the account the idea of caregivers who are necessary allies in the transition process.

This study further highlights the necessity for a common educational curriculum between paediatric and adult epileptologists to aid in an effective transition of care.

The organizational model emerged from this study will be made concrete and applied by the LICE Transition working group.

## Data Availability

Data from the literature review and Delphi rounds will be made available under request to those eligible.
